# Injuries of the Superior Extremities

**Published:** 1842-10

**Authors:** W. L. Sutton

**Affiliations:** Georgetown, Ky.


### WESTERN JOURNAL

OF

### MEDICINE AND SURGERY.

OCTOBER, 1842.

### Art. I.—*Injuries of the Superior Extremities.* By W. L. Sutton, M. D., Georgetown, Ky.

Case I. *Gunshot wound of the axilla.*—A negro boy aged about 12 years received, March 11, 1842, the load of a shot gun in the right axilla, which passed out just behind the spine of the scapula, about two inches from the glenoid cavity. There was said to have been considerable hemorrhage at first; but when Dr. Smith saw him, in perhaps half an hour, there was none of moment—no pulse in the arm, which was ascribed to the shock. It does not appear that the left arm was examined. I saw him on the 12th, when he complained but little; no pulse at the wrist, (but in the left arm, it was distinct and irritable,) sensation imperfect, but not destroyed; temperature not materially diminished, if at all; tongue furred, whitish. Gave a dose of calomel. Reapplied a bandage on the forearm and arm, and applied a bandage and compress in such a way as to act on the subclavian artery, below the humeral end of the clavicle. As he was five miles from town, and it was deemed important that he should be where he could receive strict attention, I directed him to be removed to Lexington, the place of his master's residence. I saw him no more, but was informed that he bore the transportation well. In a day or two mortification of the hand and fore-arm commenced. A well-marked separation between the dead and living parts took place above the middle of the arm; and about the 28th the arm was amputated at the line of separation. At this time, the original wound was suppurating favorably, and confident expectations of a favorable result were entertained. On the day succeeding the operation, he was seized with "spasms" (tetanus, I suppose), which recurred frequently until the 11th of April, when he died.

I should not have noticed this case, except to bring one fact to the notice of the younger members of the profession—a fact which I doubt not has been taught, but which I have no recollection of having seen or heard inculcated; which others *may* therefore be as little prepared to anticipate as I was. It is, that a sudden obliteration of the principal artery of a limb is not *necessarily* followed by a marked diminution of temperature in that limb. In support of this proposition, the only two cases bearing upon it, which have fallen under my observation, may be adduced. I refer to the foregoing case, in which I am satisfied that the axillary artery was either divided, or had its vitality destroyed by the load of shot; and to a case in which I tied the femoral artery about two inches below Poupart's ligament, in which neither the hand nor thermometer indicated an abatement of temperature.

Case II. *Arm torn off—injury to abdomen—death on the 11th day.*—Andrew, a negro aged 14, Dec. 16th, 1834, got his left arm entangled in the band, by which part of the machinery of a paper mill is propelled; he was drawn some ten feet to a partition wall, through which the band runs, with his abdomen directly against the end of a large beam, which extended through the wall, whereby his arm was torn off with scapula attached. I saw him probably in less than an hour—no pulse, surface cold; prescribed stimuli internally, and heat externally. In three or four hours the circulation improved considerably, when there being no hemorrhage, I dressed the wound by drawing the skin together as well as I could; but a small portion of the wound remained uncovered. Lint and adhesive strips completed the application to the wound itself; but it was judged proper to put a compress over the subclavian artery, to guard against hemorrhage, and another over the cavity made by the abstraction of the scapula, which were secured by a bandage, something in the manner of Desault's second for fractured clavicle.

19*th.*—Pulse has remained at 130; tongue acuminated, furred, white; skin dry; urine high colored, depositing a copious brick colored sediment; no appetite; complains little. To day he complains of tenderness in the abdominal muscles; and now for the first time I became aware of the fact before mentioned, that he was drawn with his belly against the end of the beam.

24*th.*—During the 19th he was troubled with bloody, slimy stools, which were attended with much griping. They continued three or four days, occasionally alternating with serous offensive stools. Much pain in the bowels occasionally, particularly at night. Pulse has been down to 104, but at present 112; tongue casting off fur, but has a rather dry and unpleasant appearance. The wound, where not covered by the skin, looks too dark; some imperfectly formed pus.

26*th.*—The serous, offensive stools continued. Occasionally he has a very severe pain in his bowels, lasting from 15 to 30 seconds. Considerable quantity of pus, of the color and consistence of cream, about the clavicle, and in the cavity from which the scapula was drawn. Over the clavicle the skin has sloughed, say two inches by three fourths, showing the bone dead. Pulse 124, small; tongue pale; sight failing. Died on the morning of the 27th.

*Inspection six hours after death.* Abdomen but moderately distended. Upon opening it, the small intestines were seen of a mahogany color, with here and there a spot darker than usual. They were usually of the diameter of one and a half inches; but where darker, they were also of greater diameter, and had very distinct arborizations of bloodvessels. In numerous places, although the intestine was round before it was touched, yet upon being touched, it pitted, and did not resume the cylindrical shape. The large intestines were in general healthy; but the rectum, near its commencement, was contracted for three or four inches to less than half its usual diameter. Throughout the extent of the colon there were spots which presented the same dark color and arborizations of vessels as were observed in the small intestines. At these places also the gut was irregularly pouched. The mucous coat of the small intestines, and of the spots mentioned in the large ones, was of a rather dark mahogany color. The best idea which I can give, is to refer to Horner's Pathological Anatomy, plate 1, fig. 6, and say that the color was pretty uniform, not so dark as the deepest parts of the figure, and darker than the remainder; and to pl. 1, fig. 2, accompanying Jackson's paper on Cholera, American Journal of Medical Science, vol. 12, and say that the horizontal stripe near the middle is near the color, but not deep enough. The stomach externally appeared healthy, but was not opened. The liver healthy; gall bladder filled with healthy bile. Although the mesentery did not appear unusually dry either to the sight or touch, yet it crepitated like a dry membrane on passing a scalpel through it.

The wound of the shoulder had sloughed a good deal since the last dressing. The clavicle was entirely denuded of the periosteum for three or four inches from the humeral extremity, and two thirds of its circumference. The stitches by which the skin had been drawn together, had nearly sloughed out, although they shewed no such disposition at the last dressing. The cavity over the front of the chest, and that from which the scapula was drawn, shewed no disposition to diminish. The muscles and cellular membrane beneath the clavicle were a good deal ecchymosed. The subclavian artery was filled with a coagulum from its origin to the point of laceration.

Did this boy die of the injuries done to the shoulder, or did he die of that done to the abdomen? I think the latter. He never complained of the shoulder, and it was only in the last days of his life, that sloughing shewed itself in the wound of the shoulder, whilst from the third day after the accident, there were considerable pain and great disorder in the bowels. It may be said that this disorder of the bowels supervened as a consequence of the injury done to the arm. Whilst I admit that such things do sometimes take place, I do not think that this was one of the cases. Every one will draw his own conclusions.

Case III. *Compound fracture of the humerus.*—W. K., aged 8, December 15th, 1830, fell from a horse, receiving the weight of his body principally upon the left hand. I saw him the next day. I found the humerus broken immediately above the condyles, the upper fragment forced through the skin on the fore part of the arm, to the extent of an inch and a half, which "two of the strongest men in the neighborhood had tried in vain to draw into place." The bone appearing healthy, a slight incision of the skin enabled me to reduce it. It was then put up in angular splints, and treated as directed by teachers. Nothing of note occurred, and in due time he was dismissed with a good arm.

Case IV. *Humerus fractured by a musket ball—artificial joint.*—Samuel Shannon, in the North West campaign in 1813, had a musket ball to pass through the humerus, immediately above the condyles. It would appear that no very strict treatment was pursued. After some weeks, he regularly bent his arm every day. His arm got well with the elbow stiff, and an artificial joint at the place of fracture. This artificial joint supplies the place both of the elbow joint, and of the rotatory motion of the fore-arm, in a very perfect manner, and he is able to do a good day's work at any kind of labor. I mention the case of this man, with whom I have lately become acquainted, to show what nature will sometimes do for a man, and that an artificial joint is not *always* such a bad thing.

CASE V. *Compound dislocation of the elbow—mortification—amputation—recovery.*—William Johnson, aged 13, fell from a height on his hands, Sept. 15th, 1839, by which the left elbow was dislocated, and the condyles of the humerus forced through the skin in front of the elbow joint. It was reduced and dressed in the straight position by Dr. Price.

16*th.* I saw him by invitation: much fever; pulse 134; tongue very much furred: fore-arm and hand cold, insensible to pricking, and perhaps a shade too dark; the capillary circulation not utterly destroyed, as would appear by pressing the skin, producing a white spot, which slowly disappeared.

17*th.*—Much the same, except that he was considerably delirious all night, and still continues so. Mortification has commenced upon the fore-arm. On consultation it was determined that amputation was the only chance of safety, which was immediately performed. He was in great danger for several days, notwithstanding amendment was perceptible in a few hours after the operation. After amputation, it was ascertained that the humeral artery, the two cutaneous nerves, and the veins in front of the elbow were torn asunder by the condyles of the humerus in their exit.

CASE VI. *Dislocation of the elbow—sloughing of the fore-arm—artificial joint.*—Feb. 22, 1842, I was requested to visit in consultation W. C., aged 8, when I received the following history of the case. He fell from a fence some time in December, receiving his weight on his right hand, whereby the elbow was dislocated, the condyles of the humerus being thrown forward on the fore-arm. The dislocation was reduced and a bandage and splints applied in the bent position. Upon dressing a week after, sloughing of the fore-arm had commenced, which eventually carried away all the soft parts on the outside, back, and inside of the fore-arm, nearly to the wrist, leaving the bones naked. The wrist and hand swelled very much, and mortification of the thumb and one of the fingers ensued. At present the hand and fingers are enormously swelled, but of a color suitable to that state; sensation is distinct. Necrosis of a large portion of the ulna has taken place, which portion has been removed; what remains is covered with red granulations. The radius gave way about the middle, yesterday. For about two and a half inches below the elbow joint, the soft parts remain sound; the elbow-joint flexible. He is represented as having remained in good health from the time of the accident; no fever or other disturbance. He is now in good health. The appearance of the arm presents to me the impression that the constitution is making a very considerable recuperative effort; and there being no circumstance to require immediate removal, it is agreed to wait on the efforts of nature. The arm was directed to be kept still, and in a case, so as to prevent motion of the fractured part.

*June 14th.*—I saw him accidentally; his arm in a sling without any other support; but it is said that he usually carries it in a box. The swelling of the hand has disappeared. There are two or three fistulous sores about the hand and fore-arm, and occasionally a scale of bone is discharged. The forearm is otherwise skinned over, and of good appearance; one of the branches of the radial artery has become the principal trunk. The ulna and radius have not united, but his father thinks the arm is more stiff. The elbow joint is not so flexible as it was in February. The fore-arm has shortened about two inches. No command of the fingers. The boy remains in good health.

It is too soon to say what will be the result of this case. At present the probability is that the ulna and radius will remain ununited, and that from disuse the elbow may become stiff. Yet it is possible that by proper attention both might be avoided. I certainly think that due care to prevent motion of the bones has not been used; but we dare not say that any care would have procured union.

Case VII. *Fracture of the humerus—compound fracture of the fore-arm—laceration of the external interosseal artery—arm saved.* Feb. 20, 1836, Peter, a very stout, athletic negro about 30 years old, in calendering bagging, got his hand caught between the cloth and spindle. In an instant the arm was wound round the spindle to the axilla, and his body thrown over the spindle upon the cloth, which stopped the machine.

I found him soon after in great pain; the convexity of the back of the hand destroyed, but I did not ascertain any fracture there; the bones of the fore-arm broken in three places, at the lowest of which both had lacerated the skin; the radius still projecting; no portion of bone loose; the external interosseal artery torn and bleeding; the humerus fractured at the middle. The artery was tied, and the arm and fore-arm dressed in the usual way. Three or four hours after, he was in so much pain that it was thought advisable to remove the dressings and reapply them: now he also complained of severe pain in the chest, occasioned, I suppose, by the pressure of the body against the spindle, in being thrown over it. He was now bled, and had a decided dose of opium with calomel. He was not able to lie down for a week, and continued to suffer greatly. During that time, he took several cathartics (mercurial and mixed) and such other medicines as were thought necessary to keep down inflammation, with generally an opiate at night.

Dr. Craig and myself attended this case jointly after the first dressing, and thought it necessary to dress the fore-arm very frequently. In about five months it was well (several pieces of bone having been removed in the mean time), with no deformity in the arm; the elbow joint perfect; some deformity in the fore-arm, occasioned by a neglect or inability to keep the fragments of the radius duly separated from those of the ulna: a very imperfect rotatory motion of the fore-arm; an inability to shut the hand firmly, but with sufficient use of it to enable him to do a full day's work at spinning.

Case VIII. *Fracture of the humerus—fracture of the fore-arm—mortification—amputation—recovery.*—Peyton, a young negro man, Feb. 7, 1840, got his hand caught in calendering bagging. His arm was wound up like Peter's, with two fractures of the bones of the fore-arm and one of the humerus; no laceration of the integuments. The fractures were reduced and dressed in the usual way by Drs. Craig and Price. Three days afterwards, I saw him in consultation. Mortification had commenced on the fore-arm, and was spreading rapidly up the arm; pulse about 100, not of a bad character; countenance not bad, but marked with suffering. Amputation was decided on and immediately performed. Mortification attacked the stump, but by judicious treatment was arrested, having made little progress.

Here are two cases very similar, the graver doing comparatively well, the less severe requiring amputation. Whence this difference in the result? From treatment? Not so; for I doubt not Peyton was treated as well as Peter. The accident found Peyton's system, constitution, body, or whatever term you choose to employ, less able to resist injury. Now as perhaps a majority of physicians upon seeing Peter's arm, might have thought amputation justifiable (if indeed not called for), and as few would have thought of amputating in Peyton's case, we are admonished not to have too little confidence in the powers of nature to resist injuries; at the same time to be prompt to interfere where circumstances require us to do so.

Case IX. *Fracture of the ulna—dislocation of the superior end of the radius—deformity.*—Aug. 10th, 1841, W. M., aged 10, presented himself, and I received this statement. Three weeks ago he was sitting on a fence, to which a horse was fastened, the horse becoming frightened, pulled the fence down with him on it, and hurt his arm; no distinct idea could be gathered as to the position in which he fell. Next day, when the arm was very much swelled, he was taken to a neighboring physician, who dressed the arm. Upon examination, I find the ulna has been broken at about one third its length from the elbow, and has united at a considerable angle, which, assisted perhaps by a softening of the ends of the fragments in the process of healing, has admitted a shortening of near an inch (measuring from the olecranon to the styloid process of the ulna), as compared with the other fore-arm. The superior end of the radius is thrown out on the outside of the tubercle of the humerus. For a moment I endeavored to force the button-like extremity of the radius into the lunated notch of the ulna; but a little reflection shewed me, that to effect that object, it would be necessary to lengthen the ulna, for which, again, it would be necessary to break up the union of that bone. Although neither flexion nor extension of the fore-arm could be carried to the maximum, and the rotatory motion of the hand was imperfect,, yet as the motion of the elbow joint was very considerable, without pain or inconvenience, and as some rotatory motion existed; and as some uncertainty existed as to the state of the semilunar notch and the success of reducing the head of the radius (even if the ulna were broken again), and as there was danger of an artificial joint, I decided against interference.

Was this the proper course to pursue? As I am not aware that just such a case has ever been reported, I may be allowed to offer such remarks as present themselves to my mind. Dislocation of the radius is confessedly rare. I have seen, so far as I now recollect, the details of but two cases—one by Dr. Smith, of the Pennsylvania Hospital, Med. Ex., vol. 1, p. 197, and one by Dr. Hammond, Med. Chir. Rev., vol. 32, p. 285. In both of these the luxation was backwards. So far as I know, but one other kind of luxation of this bone has been recognised, to wit, forwards. A question then may be fairly entertained, was I correct in my opinion of the nature of the accident? The very remarkable prominence projecting off from the external protuberance of the humerus, as observed by sight and touch, and the distinct concavity in the end of the bone, as very evident to the touch, left no room for doubt. But, it may be said, there was too free motion in the elbow joint. There certainly was more than I should have expected; but it must be recollected, that this state of things had lasted three weeks, and the parts concerned had made some progress in accommodating themselves to their situation. Could the radius have been reduced without breaking up the attachment of the ulna? To have enabled the glenoid cavity of the radius to come into its proper position, the ulna remaining shortened, it would have been requisite to force the radial portion of the wrist joint at least three quarters of an inch beyond the ulnar portion. If this could have been effected it would have thrown the hand towards the ulnar edge of the fore-arm, beyond any allowable position. But say that a portion of the excess of length should remain at the upper extremity. Then as soon as it was brought before the tubercle of the humerus, the motion of the elbow joint would be destroyed; or the outer condyle of the humerus forced backward, which, if it did not destroy the motion of the joint, would greatly injure it. Should the bone have been broken? Perhaps it would have been proper. But I repeat, that I do not know whether the cavity of the semilunar notch would remain three weeks in a condition fit to receive the head of the radius, or whether it would be found more or less filled with cellular substance, and of course presenting more or less difficulty in retaining the radius there when reduced.* In a recent case, what would be the best mode of proceeding? Any one perhaps could answer this as well as I could, but it appears to me, that extension and counter extension, with some force applied to the button-like end of the radius, assisted by rotation and straightening of the fore-arm, would be proper, and could scarcely fail.

Case X. *Fracture of the scaphoid cavity of the radius—fracture of the lower end of the ulna.*—Mrs. P., Septh 4th, 1838, was thrown from her carriage, and received her weight on the left hand and fore-arm, by which the scaphoid cavity of the radius was split, and the lower end of the ulna fractured just above the styloid process. I dressed the fracture in the usual way, and by attention, I had as much gratification as the most unqualified satisfaction expressed by the lady and her friends could give; yet I could see, that although there was no absolute deformity, still there was a want of symmetry, which I was much mortified to see in a lady's arm, especially as it occurred under my own management.

Case XI.—Nov. 1839, A. G., a carpenter, middle aged, fell about eight feet upon his hands, splitting the glenoid cavity of the left radius, and of course having a dislocation of the wrist. Being much dissatisfied with the usual mode of dressing in

---

* Mr. Liston, speaking of a simple luxation of the elbow joint, says: "It is almost impossible to replace the bones after three or four weeks; indeed, I have been foiled at the end of two weeks."—*Elements of Surgery*, p. 500.

such cases, and having seen a statement of Dr. J. R. Barton's mode of treating them, I determined to try it. Having the arm properly extended, I applied a roller upon the hand and fore-arm, securing a compress some third of an inch thick, and two and a half square, on the carpus and back of the hand, and a similar one on the palmar side of the radius, just above its carpal extremity, in such a way that the superior extremity of one corresponded with the inferior edge of the other. Over these I applied two thin but firm wooden splints, long enough to extend from the elbow to the points of the fingers. After a week the dressings were removed and reapplied daily pressing the fractured portion of the radius firmly so as to prevent displacement, whilst the fingers and wrist were fixed and straightened; and after ten or twelve days adding pronation and supination to these motions. At the end of treatment the wrist was found perfectly symmetrical. I hold myself under much obligation to Dr. Barton for instruction on this point.

